# Contextual modulation of primary visual cortex by auditory signals

**DOI:** 10.1098/rstb.2016.0104

**Published:** 2017-02-19

**Authors:** L. S. Petro, A. T. Paton, L. Muckli

**Affiliations:** Centre for Cognitive Neuroimaging, Institute of Neuroscience and Psychology, University of Glasgow, 58 Hillhead Street, Glasgow G12 8QB, UK

**Keywords:** primary visual cortex, cortical feedback, auditory modulation

## Abstract

Early visual cortex receives non-feedforward input from lateral and top-down connections (Muckli & Petro 2013 *Curr. Opin. Neurobiol.* **23**, 195–201. (doi:10.1016/j.conb.2013.01.020)), including long-range projections from auditory areas. Early visual cortex can code for high-level auditory information, with neural patterns representing natural sound stimulation (Vetter *et al.* 2014 *Curr. Biol.* **24**, 1256–1262. (doi:10.1016/j.cub.2014.04.020)). We discuss a number of questions arising from these findings. What is the adaptive function of bimodal representations in visual cortex? What type of information projects from auditory to visual cortex? What are the anatomical constraints of auditory information in V1, for example, periphery versus fovea, superficial versus deep cortical layers? Is there a putative neural mechanism we can infer from human neuroimaging data and recent theoretical accounts of cortex? We also present data showing we can read out high-level auditory information from the activation patterns of early visual cortex even when visual cortex receives simple visual stimulation, suggesting independent channels for visual and auditory signals in V1. We speculate which cellular mechanisms allow V1 to be contextually modulated by auditory input to facilitate perception, cognition and behaviour. Beyond cortical feedback that facilitates perception, we argue that there is also feedback serving counterfactual processing during imagery, dreaming and mind wandering, which is not relevant for immediate perception but for behaviour and cognition over a longer time frame.

This article is part of the themed issue ‘Auditory and visual scene analysis’.

## 1. Introduction

Sensory cortices receive domain-specific information through their primary afferent pathways, and information from the other senses via cortical feedback and top-down pathways [1]. These multisensory activities in sensory cortices, for example auditory signatures in primary visual cortex [2], dispel the earlier theory that multisensory processing is restricted to higher cortex. We address a number of outstanding questions as to how and why the earliest cortical area receiving input from the retina is modulated by auditory signals. One assumption is that the function of multisensory representation in visual cortex is to facilitate efficient perceptual processing for optimized behavioural responding. Now the field must achieve a comprehensive framework of this assumption that includes testable theories. We begin in this direction by discussing the relevance of internal representations in facilitatory processing across the senses, suggesting that auditory signals in visual cortex not only assist in the spatial localization of visual inputs, but that they also prepare for the type of visual input [3]. We also review a recent theory of how the active dendritic properties of pyramidal neurons could provide a mechanism for audio and visual signals to interact in V1 at a behaviourally relevant level [4]. A further assumption is that some early visual cortex activity might serve functions other than perception. When subjects sleep or engage in counterfactual daydreams, we still observe activation patterns in early visual cortex but this activation cannot be to facilitate perception. We suggest that early visual cortex is a privileged area serving internal visual representations, and that this can be modulated by other senses such as audition. We propose that multisensory processing in each sensory area should be considered as functionally discrete as each serves different gains for the brain.

## 2. Auditory signals in early visual cortex

**Figure 1.**
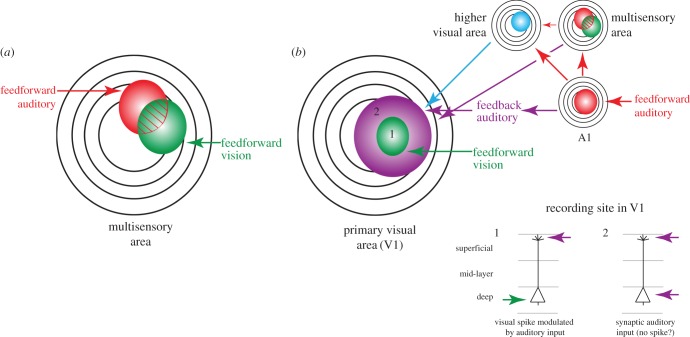
(*a*) Multisensory areas respond to audio or visual signals individually, or to a spatio-temporal overlap in audio and visual signals. (*b*) In primary visual cortex, feedforward geniculate inputs activate classical receptive fields, whereas auditory signals activate the non-classical receptive field of V1 neurons, carried by cortical feedback. Top-down auditory signals to V1 may originate directly from auditory cortex, or indirectly via extrastriate cortex or multisensory areas. (*c*) V1 responses to auditory stimulation have been investigated at different spatial and temporal resolutions (see text). It is possible that feedforward and feedback inputs arrive at individual cortical neurons [4], which can be studied in isolation using appropriate paradigms (such as visual occlusion) that mask feedforward input.

The primary function of early visual cortex is visual perception, hence why its visual properties are most established. Early electrophysiological recordings in primary visual cortex showed that in the presence of simple visual stimuli, neurons function as spatio-temporal filters that extract elementary visual features, upon which progressively higher visual areas perform increasingly complex recognition operations [5]. However, V1 neurons also respond in a more complex mode, which is seen in response to stimuli in natural vision. For example, nonlinear receptive field models using the statistics of natural stimuli are better at predicting V1 responses than a model using simple grating stimuli [6]. Currently, deep neural networks are proving effective towards the ongoing effort to predict and quantify population visual responses to natural stimuli [7,8], with the aim to characterize all levels of functional visual responses in the visual hierarchy during recognition. However, another dimension to understanding the function of the early visual system must expand beyond its role in visual recognition, because V1 is interconnected to non-visual areas and responds in the absence of visual stimulation [9], and is subject to diverse top-down influences, including motor, reward and emotional responses [10]. Descriptions of V1 responses to real-world stimulation therefore need to incorporate not only feedback and top-down inputs in the visual system, but also other endogenous inputs to V1. The classification of endogenous denotes terminations in V1 that originate from any other brain area (or within V1), but not via sensory receptors, i.e. all the non-retinal inputs [9]. We include auditory signals as a subset of cortical feedback inputs to the extra-classical receptive fields of V1 neurons (figure 1).

Crossmodal activations in sensory cortices are not of the archetypal feedforward processing whereby a sensory stimulus generates a spiking response in its dedicated sensory cortex. This makes it more challenging to realize a functional and computational description, moreover, one which is integrated with biological constraints that we also do not yet fully understand. This stimulates questions such as what kind of neural signals are we searching for? What kinds of measurement techniques are optimal? What kinds of stimuli and tasks drive these signals? We need to learn how information from discrete sensory systems reaches other sensory areas, how this information is translated across the senses, what advantage the brain gains by recruiting low-level cortex, and how networks states such as cognitive task or arousal govern this processing [1]. Although it is unclear if auditory feedback to primary visual cortex fits the profile of sensory integration seen in higher-association areas or the superior colliculus [11], anatomical and functional findings suggest that auditory signals in V1 do contribute to multisensory processing in animals and humans. Rodent, cat and monkey electrophysiology data show that V1 is modulated by auditory signals at the level of single neurons, which arrive via cortical feedback to early visual cortex. The earliest experiments, performed in the 1960s and 1970s, may have been subject to technical limitations, and for some time, there was a question over their replicability. Notwithstanding these issues, these studies implicated a role of auditory signals in visual cortex in orienting towards a visual stimulus. In areas 18 and 19 of the cat visual cortex, over 40% of neurons responded to visual and auditory stimulation, although none demonstrated a frequency-tuning curve to sound stimuli of 5–50 kHz as would be anticipated in auditory cortex. However, the location of the acoustic source modulated the responsiveness of the visual neurons [12]. The observation that visual neurons are more sensitive to where the sound originates than to the frequency of the sound has a logic coherent with the retinotopic spatial organization of the early visual system, and could be why auditory feedback to visual cortex might preferentially target peripheral regions prior to foveating the sound source [3]. Neurons responding to sounds and visual inputs have also been observed in area 17 of the cat, and they also revealed coincident receptive fields for visual and sound inputs [13]. A proportion of these neurons in cat primary visual cortex that respond to acoustic stimuli may also show a preference for specific frequencies of the sound [14]. More recently, it has been shown in awake behaving monkeys that multisensory integration of auditory and visual signals in V1 is dependent on the behavioural context. When monkeys are required to make a saccade, reduced response latencies are found in V1 neurons when the saccade is towards a visuoauditory stimulus compared with orienting gaze to a visual stimulus, if the visual stimulus is at a mid-level contrast [15].

Human primary visual cortex is also considered as multisensory in function [16], with the largest contribution to these crossmodal signals being auditory feedback. As an example of perceptual modification, subjects can visually misperceive a single flash as two flashes if the flash is paired with two beeps [17]. V1 responses that are modulated by sound reflect subjective perception and not the physically present visual stimulus; activity in the retinotopic representation of a flash is enhanced when a second flash is perceived but suppressed when two flashes are perceived as one [18]. Individuals that have smaller early visual cortices are more disposed to this flash-beep illusion [19]. Conversely, the direction of auditory motion can be misperceived if paired with opponent visual motion [20]. It seems that the more dominant feature overwrites the weaker one: temporal periodicity in audition trumps vision and visual-spatial transition trumps audition. Using human magnetoencephalography and functional magnetic resonance imaging (fMRI), it has been suggested that crossmodal interactions in auditory and visual cortices are driven by projections from the opposite sensory cortex, with conduction delays of up to 35 ms [21]. Time-resolved multisensory interactions can be studied using electrophysiological techniques that reveal early effects in sensory cortices. For example, audiosomatosensory interactions are seen in the central/postcentral scalp at approximately 50 ms after the stimulus presentation, consistent with multisensory interactions in unimodal cortices [22]. Similarly, audiovisual interactions are seen in the right parietal–occipital area at a timing of less than 50 ms, again suggesting that sensory cortices are modulated by multisensory processes [23]. Such early interactions may require attention to both modalities; approximately 50 ms after the stimulus, when both auditory and visual inputs are attended, a superadditive audiovisual integration effect is found for the P50 component [24]. Attending both auditory and visual modalities also enhances audiovisual alpha band phase synchrony compared with when subjects only attend auditory inputs [25]. Studies of multisensory convergence in human primary visual cortex and its influence on behaviour are reviewed in full in [16].

## 3. What type of information is carried by auditory signals to visual cortex?

**Figure 2.**
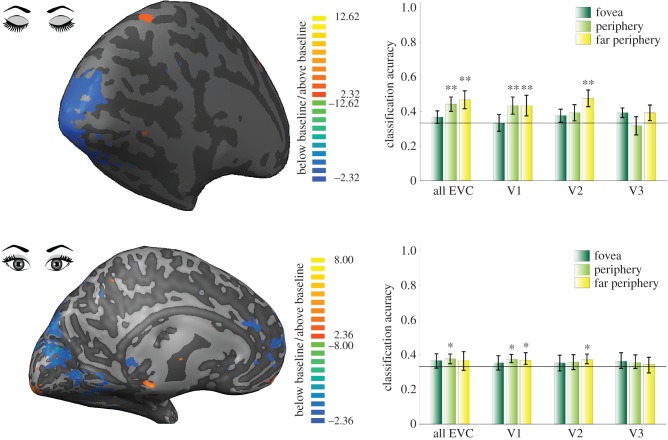
Classification performance for decoding sounds in eccentricity mapped V1, V2 and V3. The top row reports group classification accuracy from ([3], with permission) in which subjects were blindfolded. The bottom row reports group classification accuracy from a replication of this study, but with an eyes-open fixation task. Surface maps represent significant *t*-values for sound stimulation only.

Most studies demonstrating the functional effects of auditory signals in primary visual cortex have done so effectively using simple stimuli. We propose early visual cortex is also modulated by higher-level internal representations triggered by sounds. We recently showed that the semantic content of sounds and sound imagery can also be read out of early visual cortex using fMRI and multivoxel pattern analysis (MVPA) [3]. Multivoxel pattern recognition techniques in functional brain imaging search for differences in spatial patterns of brain activity in response to stimulus conditions. These patterns can be subtle and are often not detected with conventional univariate analyses that rely on averaging. We blindfolded fMRI subjects and either played complex sounds or instructed subjects to imagine them. Using MVPA, we trained a classification algorithm to learn the relationship between patterns of responses in early visual cortex and specific natural sounds. We then used this model to predict the sound labels of an independent set of patterns recorded in response to the same sounds. This approach revealed that in early visual cortex we could discriminate between complex natural sounds, particularly in peripheral and far peripheral V1 and V2 (figure 2). Sounds could also be discriminated from activity patterns when they were only imagined by the subjects, particularly in foveal and peripheral early visual areas. Sound content and sound imagery content were predictive of one another in V1 and V2, suggesting at least a partially overlapping neural code between real sound and imagined sound in visual cortex. We also tested if we could train our classifier to learn the relationship between response patterns and certain sounds, and then apply this rule to predict the category of a different set of sounds. This cross-classification approach was conceptually informative because it showed that auditory feedback information can generalize to different categorical examples, suggesting that V2 and V3 were not simply activated each time by a specific template. Such semantic or abstract sound content in primary visual cortex might be supported by feedback from higher auditory cortices and not primary auditory cortex but this remains to be tested. In addition to proposals that sound modulation of visual cortex functions to bias an organism towards the location of the sound, another function is hinted at by our ability to extrapolate across different categorical exemplars of sounds in visual cortex. Auditory modulation could bias higher visual areas to the feature content or object localization in a visual scene, potentially interacting with motor areas to orient to the source of auditory signals. Until recently, it was not known if contextual auditory information of natural scenes was also present in primary visual cortex during concurrent visual stimulation. We here replicate the findings of Vetter *et al*. [3] while subjects had their eyes open viewing uniform visual stimulation (i.e. blank fixation screen, figure 2). We found that three auditory scenes led to patterns in primary visual cortex that are specific to the scene content, and this activity is not overwritten by the simple visual stimulation with a blank, uniform grey screen with a small chequerboard fixation point. However, it remains to be seen if auditory information can be read out from early visual cortex when this is also receiving a more driving visual stimulus. Auditory scene-specific responses in early visual cortex could consist of two kinds of visual information: (i) similar visual scene information as visual feedback to V1; and (ii) similar visual scene information as feedforward visual stimulation contains when stimulated with the same semantic scene content. It remains to be tested whether auditory feedback entails any of those two sources of information. We previously compared feedforward and feedback processing in layers of V1 during visual scene processing. Using a cross-classification approach, we were able to train a classifier to discriminate visual scenes contained in the feedback signal and then apply this rule to discriminate the same visual scenes but in the feedforward condition. However, we were not able to use this same cross-classification approach to generalize from feedforward to feedback signals. We propose these results are due to the feedback signal being coarser than the fine-grained pattern in feedforward processing [26].

We suggest that our auditory scenes triggered high-level representations of visual scenes and that these high-level representations were fed back to early visual cortices, in line with theories of predictive processing [3]. In making this conclusion, we must try to eliminate attention as the sole cause of our effects. The function of visual attention is to enhance the processing of behaviourally relevant information (while inhibiting the processing of distracting features). Even in V1, this enhanced processing can be measured by an increased firing rate of neurons that have receptive fields covering task-relevant visual stimuli, these effects get stronger with increasing task difficulty [27], and this neuronal gain control can occur for spatial and feature-based responses (see [28] for review). If visual attention is selecting visual features, what of paradigms such as ours where no visual features are presented? Visual attention can act in an anticipatory way, i.e. in the absence of visual inputs [29], and feature-based attention extends to non-stimulated early visual cortex [30]. Could our sound stimuli bias attentional control of visual cortex for expected upcoming visual features? There are two reasons why we believe attention does not fully account for our effects. First, across different experiments, we have manipulated attention in various ways and found independent feedback effects [3,31]. Second, we also found attention can manipulate overall activation, but this can be different from information. The BOLD signal activation in human V1 is robustly modulated by spatial and feature-based attention [32], but univariate BOLD activation profiles are not predictive of multivariate information profiles. We previously compared BOLD per cent signal change with classifier decoding accuracy across cortical layers of human V1. During feedforward stimulation, we observed increasing BOLD activity as a function of proximity to the outer pial surface, whereas information (measured by decoding accuracy) was fairly consistent throughout all layers and peaked in middle cortical layers. Feedback-related BOLD activity was relatively stable at baseline levels across cortical depths, while information peaked in the outermost superficial layer [26]. In our auditory paradigm, we found different information patterns for our sound stimuli, but deactivation for all sounds in experiments that had no cognitive task; in those with a cognitive task we found overall unspecific activation increase in early visual cortex, suggesting our data are at least not caused by generalized attention for those stimuli. It is possible that concentrating on auditory stimulation redirects attentional processing resources concomitantly reducing the activity we see in visual cortex, and that different auditory stimuli demanded more or less attentional resources. This could explain our peripheral bias in decodability where attentional effects are strong, and incidentally enhance activity along the dorsal visual processing stream [33]. Our natural scene sound exemplars (e.g. forest sound) may have preferentially activated the dorsal stream for visually guided action or navigation though this is speculative.

Although we argue against attention as a comprehensive account of our auditory information profiles in visual cortex, potentially there is an important contribution of attention. We suggest that auditory stimuli and auditory imagery activate visual internal models, and it is for the most part this predictive processing we are detecting in early visual cortex [34–37]. Predictive coding theories would suggest that auditory scenes trigger a visual internal model leading to specific predictions about anticipated visual features, and attach a certain precision to the prediction. These predictions are transferred down to early visual cortex. In the case that expected visual inputs are not found, this mismatch leads to an error signal. The error signal propagates the cortical hierarchy if it exceeds the precision assigned to the prediction, revising the predictions at each level. At the highest level, the winning prediction is our perceptual experience. Noise can also lead to prediction errors though, and the predictions at subsequent cortical levels must not be updated on the basis of noise. Attention optimizes the precision of the error signal, and the gain is increased for reliable prediction errors, ensuring only these errors contribute to prediction modification. In this sense, attention is not incompatible with prediction [38,39]. With regards to our data, upon hearing the sounds, subjects probably activated different feature expectations and different visual-spatial configurations. This could lead to precision-weighting via attention to certain retinotopic portions of V1. When no visual input arrives, attention precision-weights the error signal, ensuring that it is not used to update the predictions at each cortical stage (assuming that our subjects had no visual perceptual experience driven by the sounds). This potential contribution of attention is not exclusive of prediction- or expectation-related effects of auditory modulation in visual cortex. Hypothetically, our sounds might have induced different arousal levels, i.e. the traffic sounds could be more alarming, leading to increased arousal compared with e.g. the forest sounds. So, although our stimuli were matched in pitch and volume, they could nonetheless trigger different arousal states. Interestingly though, arousal levels fail to induce clear decodable brain states, suggesting neural markers of arousal are less easy to find [40]. We also did not find that orthogonal task-driven effects of auditory working memory or visuospatial imagery completely inhibited auditory decoding in early visual cortex, specifically in V2. We suggest that attention-, task- and auditory-driven prediction should be considered as separate endogenous modulations of early visual cortex (see also [41]). Besides attention in early visual cortex, there are known task-dependent cognitive alterations [42,43]. Currently, the information content of our classifications is crude. It will take more follow-up experiments to test hypotheses using cross-classification and encoding modelling. Eventually, we will be able to build more realistic models that incorporate sources of information feeding from different modalities.

## 4. The anatomical constraints of auditory information in early visual cortex

There are a number of cortical regions in which multisensory processing is thought to converge. For example, electrophysiological recordings in the superior temporal sulcus of the macaque reveal neurons that respond to both visual and auditory stimuli (early studies include [42,43]). However, a number of anatomical findings contradict the early notion that multisensory processing is due to higher-level convergence of sensory signals, and instead posit an additional role of sensory cortices in multisensory integration. Perhaps the most compelling findings under this newer objective are those showing direct connections between unisensory areas in the primate [44,45], including from auditory cortex to V1. Specifically, tracer studies show that auditory input to V1 respects the eccentric organization of V1, with auditory inputs arriving more so to the region of V1 that represents the peripheral visual field [46]. This more peripheral projection of auditory signals is replicated in brain imaging studies of human visual cortex, whereby sounds can be discriminated more reliably in the periphery and far periphery of V1 than in foveal V1 cortex [3]. Cortical feedback or top-down inputs within the visual system also adhere to the retinotopic organization of V1; objects presented to the periphery lead to feedback to foveal V1 where higher-resolution object representations could assist in fine discriminations [47], whereas cortical feedback involved in scene processing arrives to the periphery of V1 [48] and extends also into the foveal cortex. The anatomical constraints of auditory signals in V1 are not only bound by the fovea versus periphery dissociation, but also by the cortical laminae termination. The auditory projections to V1 (and V2) terminate primarily in cortical layers 1 and 6, as expected by cortical feedback pathways [46]. What do these two anatomical particularities mean for the function of auditory modulation of primary visual cortex? It is possible that auditory-triggered visual feedback to V1 reaches superficial layers [26], whereas long-range feedback from auditory and auditory association cortices would terminate in deep layers of V1 [46] although cross-species similarities remain to be determined. Visual feedback could be serving predictive processing during perception and during imagery, and also counterfactual processing during other forms of imagery such as during daydreaming. Direct auditory feedback could construct predictions of lower-level sound content although it remains to be seen why primary visual cortex would receive this output, it may also be that auditory cells projecting to visual cortex are modulated themselves by auditory association cortices and that V1 actually receives this output. With regards to the fovea and periphery distinction, much anatomical and functional evidence points towards a preference of auditory signals in the periphery of V1. This type of representation could help to orient towards a visual stimulus during perception, and give spatial coordinates to mental images during auditory-induced mental imagery or semantically labelled low-resolution points in space that are tracked outside the central visual field. It has been suggested that surprising auditory inputs elicit orienting behaviour towards the source while increasing V1 sensitivity at the expected spatial location. This is supported by the superior colliculus and feedback from auditory cortex directly to V1 and via higher areas [49]. Turning to higher multisensory convergence zones, the posterior superior temporal sulcus [50] and precuneus [51] are two candidate areas to integrate audio and visual signals and to send feedback to primary sensory cortices [52]. Findings from human connectivity analyses reveal that auditory cortex is preferentially connected to the representation of the peripheral visual field in the calcarine sulcus [53,54]. However, the central visual field representation may also receive as many inputs from primary and low-level auditory cortices as the peripheral representation. Probabilistic fibre-tracking using diffusion MRI revealed fibre tracts between Heschl's gyrus and both anterior regions of the calcarine sulcus and also the occipital pole [55,56]. Lastly, we have focused on the corticocortical interactions underlying the modulation of primary visual cortex by auditory inputs, but the role of subcortical projections also needs to be elucidated.

## 5. A putative neural mechanism of auditory modulation in primary visual cortex

The function of auditory modulation in visual cortex remains an important open question: is it modulating feedforward visual processing; is it helping to construct vision or is it involved in only later stages of behavioural responding; does it contribute to our rich internal world of imagination or is its function more rudimentary than all of these ideas? Moreover, the relationship between functional auditory responses and their neural substrates in early visual cortex is not yet known. We briefly review if work on the dendritic gating of inputs to pyramidal neurons [4] could offer insights into how the cortex makes associations between visual processes and auditory-driven internal representations carried by feedback. This cellular process, for which there are plentiful rodent data, essentially entails that feedforward and feedback inputs arrive at discrete compartments of layer 5 pyramidal neurons, and that the apical dendrites that receive the feedback are critical for context-dependent gating of feedforward inputs. Long-range feedback inputs to layer 1 of sensory cortices have been found in somatosensory cortex, for example [57], and are suggested to implement top-down control on sensory processes. Feedback inputs arrive in layer 1 near to a second spike initiation zone supporting calcium spikes found in the apical tuft of layer 5 pyramidal neurons [58,59]. Providing that this second spike initiation zone receives coincident depolarization via feedback to the tufts and a back propagated spike from the cell soma, Ca^2+^ spikes are triggered at the top of the apical dendrite, and these Ca^2+^ spikes can transform a single somatic output spike into a 10 ms burst containing two to four spikes [60]. Hence, dendritic (tuft) and somatic (basal) inputs are both required for the influence of feedback in generating this cell bursting. This brief summary outlines how feedforward and feedback signals are integrated within individual pyramidal neurons [61] or more specifically how the sensory world is combined with internal representations towards perception and cognition. To the best of our knowledge, the biophysical properties of dendrites have never been studied in the context of audiovisual stimulation (i.e. recording in visual cortex with visual inputs arriving to the soma and auditory-driven feedback to the tuft dendrites) and so we have to make some assumptions. The first proposition we make is that auditory modulation of visual cortex occurs via feedback inputs (auditory to visual to early visual or directly from auditory to early visual) onto the apical tuft dendrites (of layer 5 pyramidal neurons) in layer 1 of early visual cortex. Such feedback-modulated circuits for perception have been observed between mouse motor and somatosensory cortices [62] suggesting that longer-range connections across auditory and visual cortices could conceivably support a similar process. Other questions concern the presence of such a mechanism in primates (especially humans); answers will rely on advancing technologies and suitable paradigms [63]. Another question concerns the readout of top-down signals in visual cortex in the absence of feedforward input; if this associative mechanism only serves conscious perception, which mechanism supports purely feedback representations (blindfolded, dreaming, imagining)? fMRI is sensitive to dendritic processing, meaning it is not inconceivable that paradigms removing feedforward input are still detecting the internal representations carried by feedback as outlined in [4].

## 6. The singularity of primary visual cortex

Contrary to early functional descriptions of V1 that were based only on visual processes, we have described how auditory signals are among the wide-ranging endogenous (non-sensory) inputs that can modulate processing in V1 [10,64]. Supplementary to V1's role in feedforward vision during which the external world is imprinted with high spatial resolution onto veridical receptive fields, we endorse the inverse process too, that V1 acts as a screen where the brain projects its internal world [65]. In this context, V1 is a privileged area serving lower-resolution, internally generated visual representations. This notion rests on the ability to measure how primary visual neurons adapt their response under the influence of diverse internal signals [9]. One question resulting from this hypothesis is how feedforward and feedback streams coincide in V1 during eyes-open awake states (i.e. when there is feedforward input). Intuitively, the correspondence between feedback and feedforward representations must be coarse to fine, respectively. Feed-forward representations in V1 are of a high spatial resolution, but feedback to V1 is less spatially precise [26] and contains higher-level (more abstract) representations of stimulus spaces [66]. This computation of sensory inputs with top-down influences depends on the instruction of the top-down signal; for example, feedback can reconstruct an absent feedforward stimulus [67,68], enhance an illusory feedforward input [69] or suppress a predictable stimulus [70]. We do not know yet how these two streams of representation are combined in V1, but assume that both are constrained by retinotopy. Another challenge is to conceptualize and test how auditory signals can modulate internally generated visual representations. The association of a sound and an image is intuitive in higher cortex representing high-level features; for example, the fusiform face area is structurally connected to voice-sensitive areas in the superior temporal sulcus [71]. However, for early visual cortex, the scenario of sound-driven modulation is more puzzling. For simple stimuli, sounds induce hyperpolarization in supra- and infragranular layers of mouse primary visual cortex through an inhibitory subcircuit in the deep layers of V1 [2]. This sound-mediated inhibition in V1 leads to reduced visual responses during concurrent audio and visual stimulation, indicating that salient auditory stimuli inhibit the representation of distracting visual stimuli in visual cortex via direct feedback from A1 to V1. However, what additional operations do auditory stimuli lead to in visual cortex; they cannot merely change the gain to feedforward visual inputs, because auditory information can be decoded from V1 in the absence of visual stimulation. Moreover, we know that the feature space is more complex than tones and oriented bars, especially in humans [3]. Auditory-driven modulation of primary visual cortex in this richer context of human perception and cognition could operate via feedback to V1 in at least the following modes: (i) the stimulation of visual imagery (in higher visual areas); (ii) the stimulation of visual predictions (in higher visual areas); (iii) feedback from multisensory convergence areas; and (iv) direct monosynaptic connections from auditory cortex. The distinction between auditory-induced visual imagery and auditory-induced visual prediction is somewhat subtle but has implications for behaviour. Both could induce an identical internal representation of a visual stimulus that is fed back to visual cortex. The most important difference, however, is that predictions will be veridical, generating an internal model as best as possible predicting incoming information, whereas visual imagery can be counterfactual [72]. To illustrate, imagine while on holiday reading a book on the beach in which the author mentions Janáček's Sinfonietta, creating an auditory-triggered visual image of an orchestra. This mental construct contradicts the perceptual environment making imagery in this case counterfactual. At the same time, you continue to perceive the perceptual environment of your book and the beach. Should you hear a seagull, however, your brain uses predictions to explain away the moving white object in the sky. In sum, auditory-induced visual representations fed back to V1 may or may not be similar when those representations are driven by imagery or prediction. Both could recruit compressed information from memory in the same manner and use primary visual cortex to restore the spatial information. Both would also stipulate that V1's receptive fields are flexible and adaptive, and could support a dream-like representation space in addition to its veridical receptive fields. The organism can use counterfactual imagery to play out scenarios in its mind to test consequences and make decisions. Predictions, on the other hand, follow a different function, they provide the message that it is safe for daydreaming, nothing alarming or unexpected is happening.

## 7. Conclusion

In the visual system, the effects of cortical feedback on primary visual cortex are varied, and mostly studied in the context of how they modify sensory processing and help to construct perception. Anatomical, functional and physiological characteristics of cortical feedback have motivated computational models of predictive coding and belief propagation in the visual system [73–75]. We propose that auditory feedback to V1 may activate visual predictive codes; however, we also discuss the possibility that cortical feedback serves internal counterfactual model representation. Counterfactual representations exist routinely during mental time travel, cognitive tasks and dreaming. The brain might have evolved a counterstream of veridical and counterfactual representations throughout the cerebral cortex. These counterstreams may be integrated in the case of multisensory processing.

## Data accessibility

Data are either part of or a replication of a previous publication (Vetter, Smith, Muckli, 2014, *Curr. Biol.* http://dx.doi.org/10.1016/j.cub.2014.04.020 reproduced with permission). New data are stored in anonymized format on a data server on Institute of Neuroscience and Psychology, University of Glasgow and can be distributed upon request (CCNi project no. 0167).
